# In vivo monitoring of *Lactiplantibacillus plantarum* in the nasal and vaginal mucosa using infrared fluorescence

**DOI:** 10.1007/s00253-022-12121-8

**Published:** 2022-08-24

**Authors:** Sergio Silva-Bea, Mónica Francisco-Tomé, Jorge J. Cabrera-Alvargonzález, Carmen Potel, Maximiliano Álvarez, Sonia Pérez, Benito Regueiro, Maria P. Cabral

**Affiliations:** 1 Group of Microbiology and Infectious Diseases, Galicia Sur Health Research Institute (IISGS), Vigo, Spain; 2 Microbiology Service, University Hospital of Vigo, Vigo, Spain

**Keywords:** In vivo imaging, Infrared fluorescent protein, Lactic acid bacteria, *Lactiplantibacillus plantarum*, Mice, Mucosal administration

## Abstract

**Abstract:**

Lactic acid bacteria (LAB) of the genus *Lactiplantibacillus* have been explored as potential mucosal vaccine vectors due to their ability to elicit an immune response against expressed foreign antigens and to their safety. However, tools for monitoring LAB distribution and persistence at the mucosal surfaces are needed. Here, we characterize *Lactiplantibacillus plantarum* bacteria expressing the infrared fluorescent protein IRFP713 for exploring their in vivo distribution in the mucosa and potential use as a mucosal vaccine vector. This bacterial species is commonly used as a vaginal probiotic and was recently found to have a niche in the human nose. Three different fluorescent *L. plantarum* strains were obtained using the nisin-inducible pNZRK-IRFP713 plasmid which contains the *nisRK* genes, showing stable and constitutive expression of IRFP713 in vitro. One of these strains was further monitored in BALB/c mice using near-infrared fluorescence, indicating successful colonization of the nasal and vaginal mucosae for up to 72 h. This study thus provides a tool for the in vivo spatiotemporal monitoring of lactiplantibacilli, allowing non-invasive bacterial detection in these mucosal sites.

**Key points:**

*• Stable and constitutive expression of the IRFP713 protein was obtained in different L. plantarum strains.*

*• IRFP713* ^*+*^ *L. plantarum 3.12.1 was monitored in vivo using near-infrared fluorescence.*

*• Residence times observed after intranasal and vaginal inoculation were 24–72 h.*

**Graphical abstract:**

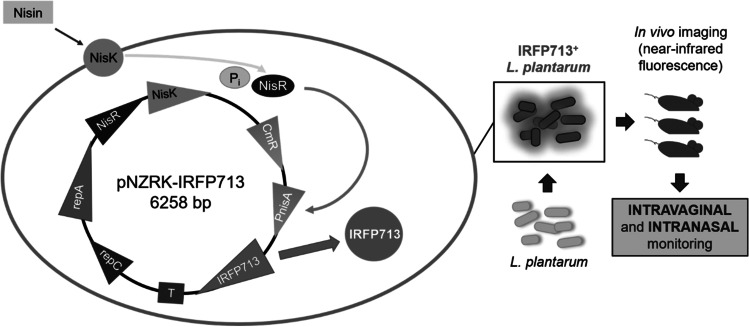

**Supplementary Information:**

The online version contains supplementary material available at 10.1007/s00253-022-12121-8.

## Introduction

Lactiplantibacilli and other lactic acid bacteria (LAB) are non-pathogenic microorganisms that have long been used in the food industry (Azam et al. 2017; Behare et al. 2020; Li et al. 2017) and as health-promoting functional food ingredients (Gareau et al. 2010). The long history of safe use of many species of *Lactiplantibacillus* gave them GRAS (generally-recognized-as-safe) status (Salminen et al. 1998). Lactiplantibacilli have also gained increasing interest as vectors for the delivery of prophylactic molecules through the intranasal, oral or vaginal mucosa (LeCureux and Dean 2018; Wang et al. 2016) as they tend to induce high levels of systemic and mucosal antibodies against the expressed foreign antigens (Wells and Mercenier 2008). In this regard, accumulating evidences show that *Lactiplantibacillus plantarum* is an effective vaccine vector for gaining mucosal immunity against several infectious diseases (Kuczkowska et al. 2016; Shi et al. 2016; Wang et al. 2019), also likely to be a desirable approach to fight SARS-CoV-2, which is mainly transmitted via the mucosal route (Taghinezhad-S et al. 2021).

*L. plantarum* is part of the healthy vaginal flora and has been proposed for the treatment and prevention of genitourinary infections, gaining importance as probiotics. Local use of these microorganisms results in the restoration of vaginal microbiota, interfering with the colonization and growth of potential pathogens such as *Candida albicans* and *Gardnerella vaginalis* (Kang et al. 2018; Palacios et al. 2016; Vicariotto et al. 2014). This bacterium has also been found to be enriched in the healthy human nose and nasopharynx (de Boeck et al. 2020), which suggests its beneficial effects in this ecosystem, as previously observed for lactiplantibacilli genera (Rosas-Salazar et al. 2018). In this regard, the ability to track *L. plantarum*, as well as other lactiplantibacilli, in the vaginal and nasal mucosa of live animals would thus provide helpful information of their distribution and residence times, assessing their value as probiotics or recombinant vaccine vectors for intravaginal and intranasal delivery.

In vivo optical imaging is a non-invasive method comprising the use of bioluminescent and fluorescent reporters and probes to investigate molecular events and monitor disease progression in animal models. This tool is particularly useful for the study of persistence and spatiotemporal distribution of bacteria in living animals, as well as for monitoring bacterial infections (Romero Pastrana et al. 2018). Such imaging can be performed either using endogenous, genetically encoded luciferase (bioluminescence) (Doyle et al. 2004) or green fluorescent protein (GFP) (Naninck et al. 2021) or using exogenous fluorescent tracers that target and/or illuminate bacteria (Van Oosten et al. 2015). Fluorescence has several advantages in comparison to bioluminescence as it does not require the administration of luciferin, yields brighter signal, and is more appropriate for combining with microscopy or flow cytometry (Calvo-Álvarez et al. 2015; Choy et al. 2003).

Some fluorescent proteins, like GFP (Geoffroy et al. 2000) and mCherry (Mohedano et al. 2019) have been expressed in *L. plantarum* and used for the study of intestinal colonization of mice. However, using these fluorophores usually involve lower sensitivity and lower signal to noise ratio due to shorter excitation/emission wavelengths (overlapping with high hemoglobin absorption) and tissue autofluorescence (Choy et al. 2003). The animals are thus sacrificed for culturing inoculated bacteria. To overcome this limitation, the infrared fluorescent protein IRFP713 (GenBank accession number AEL88490) with absorption and emission maxima in the near infrared region—690/713 nm—was designed and expressed constitutively in bacteria (Filonov et al. 2011). This fluorescent protein was obtained by mutagenesis of RpBphP2 from *Rhodopseudomonas palustris*, requiring heme catabolic product biliverdin as a covalently bound exogenous chromophore (Filonov et al. 2011). Using near-infrared fluorescence reduces background tissue autofluorescence, which is minimal in the 700–1,000 nm region (Frangioni 2003). Berlec et al. (2015) have previously expressed IRFP713 in *Lactococcus lactis*, *L. plantarum*, and *Escherichia coli* in order to study bacterial persistence and their precise location in the gastrointestinal tract of mice via inducible or constitutive expression system. However, no information was provided regarding the convenience of this in vivo approach for imaging bacteria on mucosal surfaces other than the intestine.

In this study, we report the expression of IRFP713 in three prototype strains of LAB *L. plantarum*, comparing their fluorescence levels in relation to bacterial density and during the long-term storage. One of these strains, *L. plantarum* 3.12.1, was further inoculated in mice through the intranasal and intravaginal routes and monitored in vivo using the In Vivo MS FX PRO system (Bruker), coupling X-ray, and near-infrared fluorescence imaging. Results show stable and long-term constitutive expression of IRFP713, providing a tool for non-invasive bacterial detection and monitoring of lactiplantibacilli in the nasal and vaginal mucosa.

## Materials and methods

### Bacterial strains and growth conditions

The bacterial strains used in this study are listed in Table 1. *L. plantarum* 3.12.1 was obtained from the vaginal swab of a premenopausal woman and identified by MALDI-TOF and 16S rRNA sequencing (Fig. S1). Lactiplantibacilli strains were grown at 37 °C in De Man, Rogosa and Sharpe (MRS) medium without aeration. *Escherichia coli* was grown at 37 °C in Luria–Bertani (LB) medium with aeration. Chloramphenicol (Cm) was added at a concentration of 10 µg mL^−1^, when appropriate for plasmid selection. MRS was from Scharlau (Barcelona, Spain). LB, Cm, biliverdin HCl, and nisin were from Merck (Darmstadt, Germany).

**Table 1:** Strains, plasmids, and primers used in this study

Strain or plasmid	Relevant features	Source or reference
*L. plantarum* strains		
ATCC 8014	Wild type	CECT
NCDO 1193	Wild type	CECT
3.12.1 (CECT 30353)	Obtained from a vaginal swab of a premenopausal woman	This study
*E. coli*		
MC1061	recA^+^, Str^R^ and Zeo^R^	CECT
Plasmids		
pNZRK-IRFP713	pNZ8148 derivative (P*nisA*, Cm^R^, nisin-controlled expression) containing *irfp713*, *nisR*, and *nisK* genes	Berlec et al. 2015^27^
Primers		
pNZ-MCS-F	CGTTCGAAGGAACTACAA	This study
pNZ-MCS-R	GCAACACGTGCTGTAATT	This study

^*CECT*^^, Spanish Type Culture Collection; *Str*, streptomycin; *Zeo*, zeocin; *Cm*, chloramphenicol^

### Construction of IRFP713-expressing *L. plantarum*

**Fig. 1:**
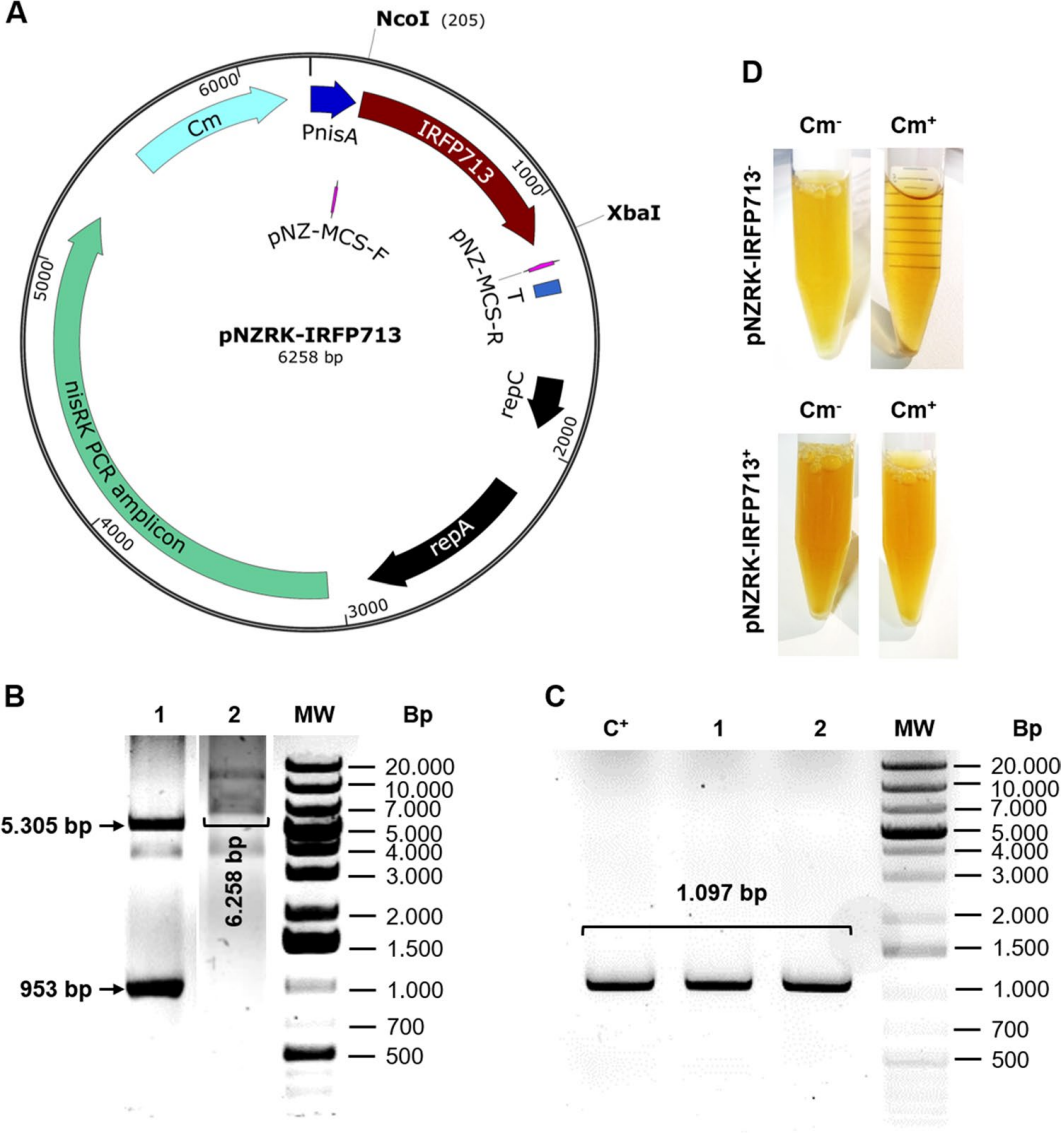
Construction of IRFP713-expressing *L. plantarum***. A** Map of pNZRK-IRFP713 plasmid, a pNZ8148 derivative (Cm^R^, P*nisA* for nisin-controlled expression, replication genes *repA* and *repC*, and a transcriptional terminator T after the multiple cloning site) in which *irfp713*, *nisR*, and *nisK* genes were introduced. The replicon of pNZ8148 came originally from *Lactococcus lactis* pSH71, allowing replication in many Gram-positive bacteria such as *L. plantarum*. pNZ-MCS-F and pNZ-MCS-R are primer binding sites used for screening pNZRK-IRFP713 transformants. *NcoI* and *XbaI* restriction sites are indicated. **B** Lane 1, pNZRK-IRFP713 plasmid digested with *NcoI* and *XbaI*; lane 2, undigested pNZRK-IRFP713; MW, molecular weight marker. **C** PCR screening of IRFP713^+^ *L. plantarum* NCIMB 8014 transformants. Transformants were screened for the presence of pNZRK-IRFP713 with primers pNZ-MCS-F and pNZ-MCS-R, which amplified a 1.097 bp region containing the *irfp713* ORF. Lanes: C^+^, pNZRK-IRFP713 plasmid used for transformation; MW, molecular weight marker. **D** Phenotypic screening of *L. plantarum 3.12.1* transformants. Upper panels: wild-type *L. plantarum* shows sensitivity to Cm-containing medium (Cm^+^). Lower panels: pNZRK-IRFP713^+^ *L. plantarum* 3.12.1 displays a Cm-resistant phenotype

IRFP713 open reading frame was previously cloned into different expression plasmids by Berlec et al. (2015) and expressed in *Lactococcus lactis*, *L. plantarum* and *E. coli*. The plasmid construct used to express IRFP713 in *L. plantarum*—pNZRK-IRFP713—was derived from pNZ8148 (De Ruyter et al. 1996; Kuipers et al. 1998), in which the *irfp713* gene was cloned, along with copies of *nisR* and *nisK* genes, which enables induction with nisin (Mierau and Kleerebezem 2005). pNZRK-IRFP713 was transformed into *E. coli* MC1061 competent cells by electroporation. Transformants were selected in LB agar plates containing chloramphenicol and PCR-screened using primers pNZ-MCS-F and pNZ-MCS-R. pNZRK-IRFP713 was purified from *E. coli* cultures using the NucleoSpin Plasmid EasyPure kit (Macherey–Nagel, Dueren, Germany) and verified by *NcoI* and *XbaI*-digestion. Taq DNA polymerase was from Roche (Mannheim, Germany). Restriction enzymes were from Thermo Fisher Scientific (Waltham, USA). *L. plantarum* strains ATCC 8014 and NCDO 1193 were transformed with pNZRK-IRFP713 plasmid according to Alegre et al. (2004). *L. plantarum* 3.12.1 was transformed using the *Procedure 2* described in Aukrust et al. (1995). *L. plantarum* transformants were screened in MRS and MRS agar plates containing chloramphenicol and PCR-screened using primers pNZ-MCS-F and pNZ-MCS-R. The plasmid map, *NcoI* and *XbaI-*restriction digestion of pNZRK-IRFP713, and screening of *L. plantarum* transformants are shown in Fig. 1. pNZRK-IRFP713, pNZ-MCS-F and pNZ-MCS-R are listed in Table 1.

### Visualization and measurement of IRFP713 fluorescence in vitro using microscopy

**Fig. 2:**
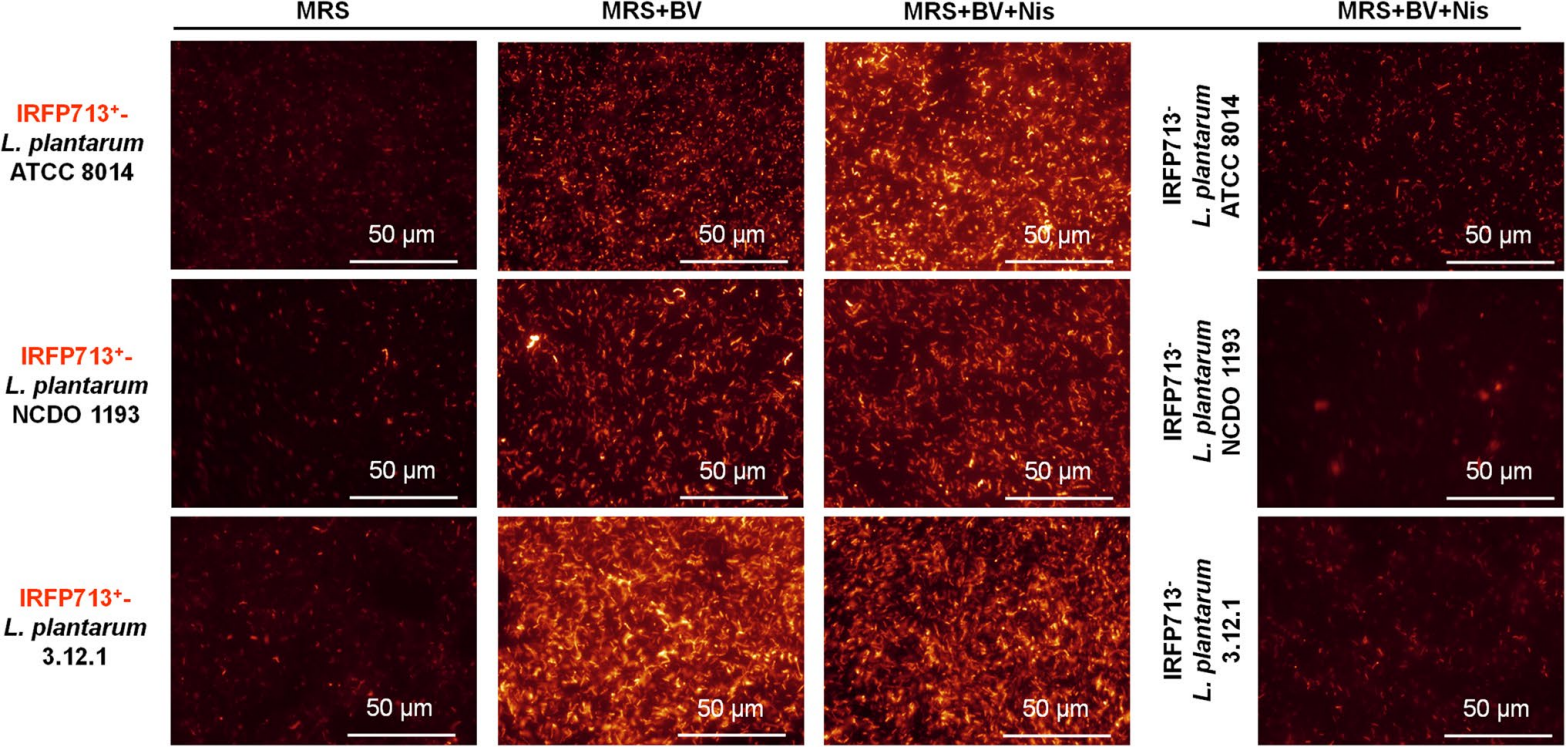
Images of IRFP-expressing *L. plantarum* observed using a fluorescence microscope. Left panels: fluorescence of IRFP713-expressing *L. plantarum* ATCC 8014, NCDO 1193 and 3.12.1 in MRS, MRS supplemented with biliverdin (BV) and MRS plus BV and nisin (Nis). Right panels: micrographs of wild-type *L. plantarum* ATCC 8014, NCDO 1193, and 3.12.1 are shown for comparison

**Fig. 3:**
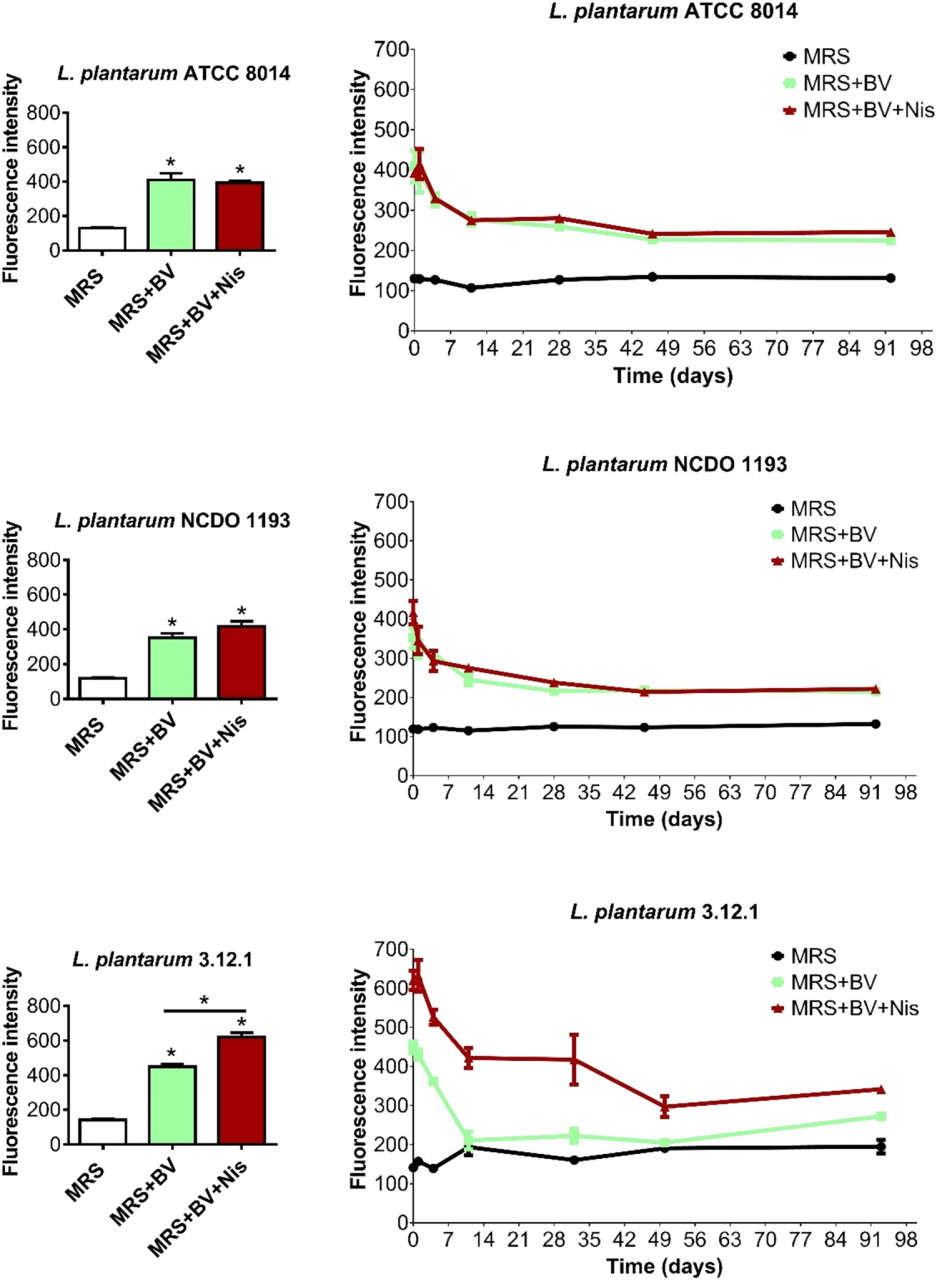
Quantification of infrared fluorescence produced by IRFP713-expressing *L. plantarum* strains using fluorescence microscopy. Fluorescence intensity of IRFP713-expressing *L. plantarum* ATCC 8014, NCDO 1193, and 3.12.1 in MRS, MRS supplemented with biliverdin (BV), and MRS plus BV and nisin (Nis). Left panels: fluorescence intensity measured immediately after culturing the bacteria. *P < 0.05 (Student’s *t* test) compared to MRS or indicated otherwise. Right panels: fluorescence intensity obtained during several days using the same bacterial cultures stored at 4 °C. The data shown are mean ± SEM of 3–4 biological replicates

Overnight cultures of pNZRK-IRFP713-harboring *L. plantarum—*IRFP713^+^-*L. plantarum* ATCC 8014, NCDO 1193, and 3.12.1 (MRS agar supplemented with Cm)—were used to inoculate 5 mL of MRS supplemented with Cm, MRS supplemented with Cm and biliverdin, and MRS supplemented with Cm, biliverdin, and nisin. Wild-type IRFP713^−^-*L. plantarum* strains (MRS agar) were used to inoculate 5 mL of MRS supplemented with biliverdin and nisin. When indicated, biliverdin and nisin were provided in the bacterial growth medium at 15 µg mL^−1^ and 100 ng mL^−1^, respectively. The bacterial suspensions were incubated overnight at 37 °C, 200 rpm, yielding OD_600nm_ values of 2.5–3.1, centrifuged (20.000 g, 5 min), resuspended in 0.3 M sucrose and stored at 4 °C until use. Aliquots of 10 µL of these cell suspensions were observed using a LEICA DMI6000 B inverted microscope coupled with 620/60 excitation and 700/75 suppression filters, using a 63 × objective and controlled with the Leica LAS X software. Exposition was set at 930.62 for visualizing and measuring the fluorescence intensity. Fluorescence microscopy images were obtained immediately after culturing (Fig. 2) and used to determine the fluorescence intensity of IRFP713-expressing *L. plantarum* strains (Fig. 3, left panels). The fluorescence intensity of IRFP713-expressing *L. plantarum* strains were also monitored until day 92–93, using the bacterial aliquots stored at 4 °C (Fig. 3, right panels).

### Viability of wild-type and IRFP713-expressing *L. plantarum*

Overnight cultures of wild-type (MRS agar) and pNZRK-IRFP713-harboring *L. plantarum* strains (MRS agar supplemented with Cm) were used to inoculate 5 mL of fresh MRS (and MRS containing Cm) medium supplemented with biliverdin (15 µg mL^−1^) and nisin (100 ng mL^−1^), incubated overnight (37 °C, 200 rpm, yielding OD_600nm_ values of 2.5–3.1), centrifuged (20.000 g, 5 min), resuspended in 0.3 M sucrose, and stored at 4 °C. The viability of the bacteria contained in these aliquots was determined until day 185 by plating in MRS agar, overnight incubation and CFU counting.

### In vitro measurement of IRFP713 fluorescence using a multimodal reader

Overnight cultures of pNZRK-IRFP713-harboring *L. plantarum* (MRS agar supplemented with Cm) were used to inoculate 5 mL of fresh MRS medium supplemented with Cm, MRS supplemented with Cm plus biliverdin, and MRS supplemented with Cm plus biliverdin plus nisin. The bacterial suspensions were incubated overnight at 37 °C, 200 rpm, diluted 1:100 in fresh medium, dispensed (200 µL per well) in a U-bottom, 96-well suspension culture plate (GreinerBioOne) and incubated in a BMG FLUOstar Omega multimodal reader (Biotek). Agitation was set to 200 rpm with incubation at 37 °C. OD_600nm_ and fluorescence (excitation: 620 nm; emission: 700 nm; number of flashes per well: 20) measurements were taken automatically each 30 min during 36 h. To avoid evaporation and condensation, the plates were sealed with transparent, hydrophobic, and gas permeable plastic films (Breathe-Easy®, Sigma-Aldrich) during incubation and measurement. OD_600nm_ and fluorescence measurements were performed in different experiments.

### In vivo imaging of IRFP713-expressing bacteria in mice

Eleven, 8- to 10-week-old female BALB/c mice were bred in the animal facility at *Centro de Investigacións Biomédicas (CINBIO),* Universidade de Vigo, in pathogen-free conditions, with food and water ad libitum. Alfalfa-free diet was used to minimize background fluorescence at least 4 days before the start of experiments. Overnight cultures of wild-type (MRS agar) and pNZRK-IRFP713-harboring *L. plantarum* 3.12.1 (MRS agar supplemented with Cm) were used to inoculate 25 mL of MRS medium supplemented with biliverdin plus nisin and MRS medium supplemented with Cm plus biliverdin and nisin, respectively. The bacterial suspensions were incubated overnight at 37 °C, 180 rpm, yielding OD_600nm_ values of 1.5–1.7, centrifuged (20.000* g*, 10 min), washed 2 times with 0.3 M sucrose, and finally resuspended in 100 µL 0.3 M sucrose. One µL of each bacterial suspension was used to determine fluorescence using in vitro microscopy and for CFU counting. After being anesthetized with isoflurane (Forane), mice were inoculated with the bacterial suspensions: three mice were inoculated intranasally with 20 μL, and four mice were inoculated intravaginally with 50 μL. Control mice were administered saline: two mice were inoculated intranasally with 20 μL, and two mice were inoculated intravaginally with 50 μL. Immediately prior to the bacterial inoculation, mice were carefully shaved on the perinasal and perivaginal areas, respectively. The In Vivo MS FX PRO system (Bruker) was used for fluorescence imaging and X-ray of mice. X-ray and fluorescence captions were obtained for each mouse (emission/excitation wavelengths 650/700 nm) and for the tubes containing the bacterial suspensions of wild-type *L. plantarum* 3.12.1 and IRFP713-expressing *L. plantarum* 3.12.1, used to inoculate mice. The region of interest (ROI) was manually set in the fluorescence caption (RGB spectrum) of each mouse and used to overlay the X-ray caption using the Image Mask Overlay function. The ROI boundaries were previously defined to display fluorescence derived from IRFP713 by subtracting the background fluorescence of the wild-type strain to the specific fluorescence derived from the IRFP713-expressing *L. plantarum* 3.12.1 bacterial suspension. Images were analyzed using the Molecular Imaging Software from Bruker.

## Results

### Characterization of infrared fluorescent *L. plantarum*

Fluorescence microscopy images (Fig. 2) were obtained after culturing IRFP713^+^-*L. plantarum* ATCC 8014, NCDO 1193, and 3.12.1 in MRS supplemented with Cm, MRS supplemented with Cm and biliverdin, and MRS supplemented with Cm, biliverdin and nisin. Wild-type IRFP713^−^-*L. plantarum* strains were cultured in MRS supplemented with biliverdin and nisin and used for comparison. Fluorescence intensity of IRFP713-expressing *L. plantarum* strains were also determined immediately after culturing and until day 92–93, using the LEICA DMI6000 B inverted microscope (Fig. 3). Immediately after culturing the bacteria, the fluorescence intensity of the three IRFP713-expressing *L. plantarum* strains were significantly higher in the presence of biliverdin than that of the background fluorescence in MRS (Fig. 3). No significant differences were found in the fluorescence intensities of IRFP713-expressing *L. plantarum* strains when nisin was added, except for *L. plantarum* 3.12.1, which showed significantly higher fluorescence intensity after cultivation in nisin-supplemented media. A drop of fluorescence intensity was observed until day 28 for both *L. plantarum* ATCC 8014 and NCDO 1193 in the presence of biliverdin, but the latter remained relatively stable over the course of 92 days at 4 °C, and still higher than the background fluorescence in MRS. When using fluorescence microscopy, the highest fluorescence intensity was detected in *L. plantarum* 3.12.1. In this strain, fluorescence intensity dropped until day 50 in the presence of nisin, remaining higher than the background fluorescence afterwards. In the absence of nisin, the drop on fluorescence intensity was noted after 11 days of storage, until being almost similar to that of the background culture. High bacterial viability was enumerated during the first 60 days of storage for the three *L. plantarum* strains (Fig. S2).

**Fig. 4:**
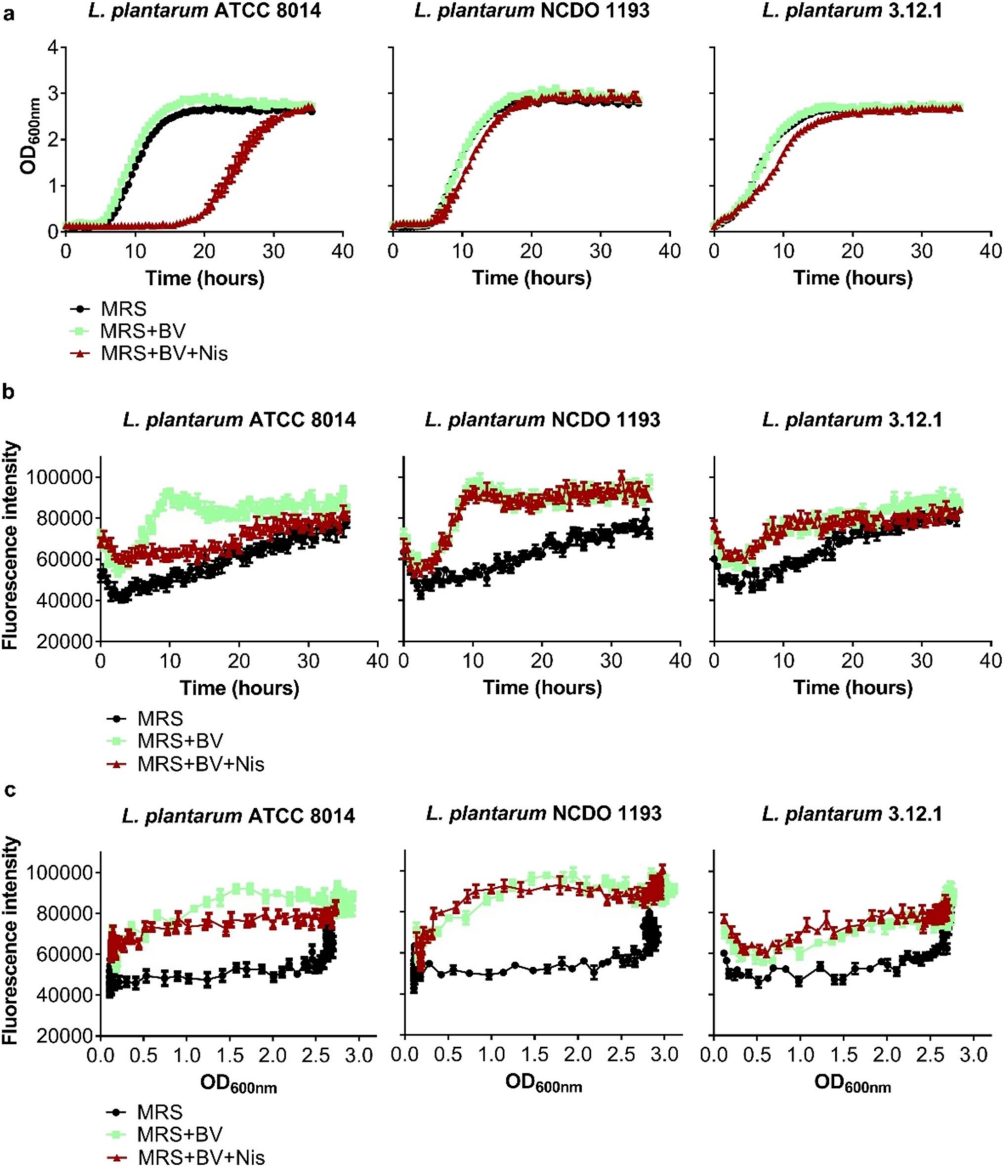
Infrared fluorescence produced by IRFP-expressing *L. plantarum* during bacterial growth. **a** Growth of IRFP713-expressing *L. plantarum* ATCC 8014, NCDO 1193, and 3.12.1 in MRS, MRS supplemented with biliverdin (BV), and MRS plus BV and nisin (Nis). **b** Fluorescence intensity of IRFP713-expressing lactiplantibacilli along the time. **c** Fluorescence intensity of IRFP713-expressing lactiplantibacilli as a function of optical density. **a–c** The data shown are mean ± SEM of 6 biological replicates

Despite successful production of IRFP713 in all three *L. plantarum* strains, slightly different fluorescence intensities to the cell concentration were observed when using a multimodal reader for measuring fluorescence (Fig. 4). In this case, the highest fluorescence intensity was detected in *L. plantarum* NCDO 1193, whereas *L. plantarum* ATCC 8014 and 3.12.1 strains yielded similar maximum fluorescence intensities (Fig. 4b and c). In contrast to *L. plantarum* NCDO 1193 and 3.12.1, in which no differences in growth were observed when using supplemented MRS for culturing, *L. plantarum* ATCC 8014 showed a more extended lag phase in the presence of nisin (Fig. 4a). For the tested conditions, *L. plantarum* ATCC 8014 and NCDO 1193 grew exponentially until *t* = 15 h and OD_600nm_ = 2.46 and 2.65 (in MRS), respectively; *L. plantarum* 3.12.1 grew exponentially until *t* = 11 h and OD_600nm_ = 2.29. During exponential growth, maximum fluorescence was registered at *t* = 10 h for *L. plantarum* ATCC 8014 (MRS + BV), *t* = 11 h for NCDO 1193 (MRS + BV), and *t* = 10.5 for 3.12.1 (MRS + BV + Nis) strains. During exponential and the early stationary phases, fluorescence intensity of the three IRFP713-expressing *L. plantarum* strains were higher in the presence of biliverdin than that of the background fluorescence in MRS (Fig. 4), but no increase in fluorescence was found when nisin was added. During late stationary phase, fluorescence levels observed for cells growing in MRS were more similar to those noted for bacteria cultured in biliverdin, which suggests unspecific signal derived from the accumulation of non-viable cellular debris and metabolic waste products.

### In vivo imaging of mice following intranasal and intravaginal inoculation of IRFP713-expressing* L. plantarum*

IRFP713-expressing *L. plantarum* 3.12.1 was further inoculated in BALB/c mice through the intranasal and intravaginal routes and monitored in vivo using the In Vivo MS FX PRO system. Bacteria were kept at 4 °C for 3–4 days prior to administration to ensure stable fluorescence during the in vivo experiments.

**Fig. 5:**
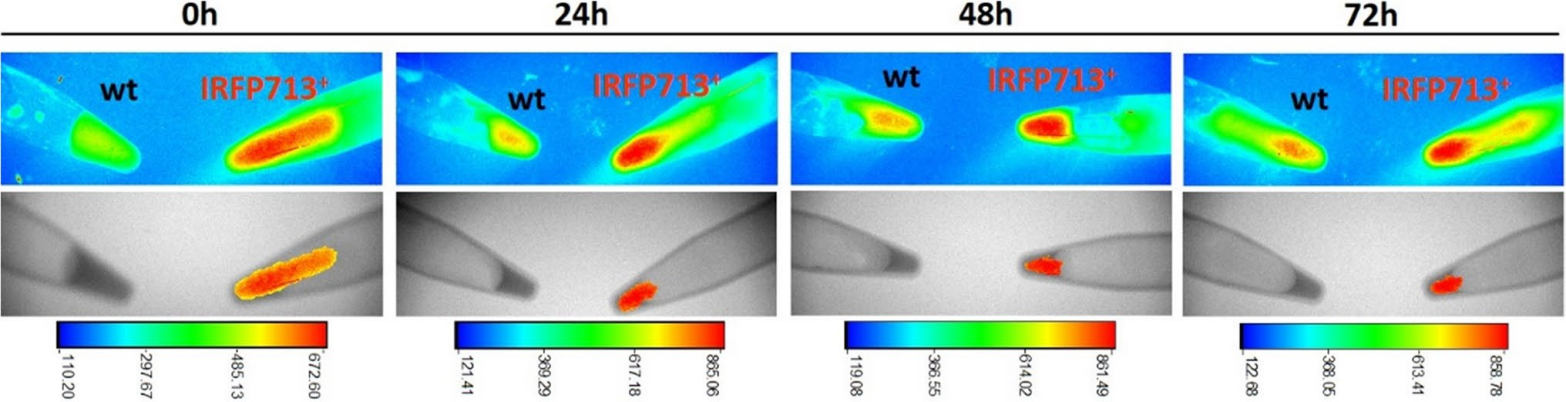
In vitro imaging of infrared fluorescence produced by IRFP713-expressing *L. plantarum* at different time points. Upper panels: fluorescence images of IRFP713-expressing *L. plantarum* 3.12.1 and wild-type 3.12.1 (2 × 10^11^ CFU mL^−1^), obtained at emission/excitation wavelengths 650/700 nm. Lower panels: X-ray images with IRFP713-derived signal fluorescence overlay. Color bars indicate fluorescence intensity. Images were obtained using Bruker In Vivo (MS) FX PRO

**Fig. 6:**
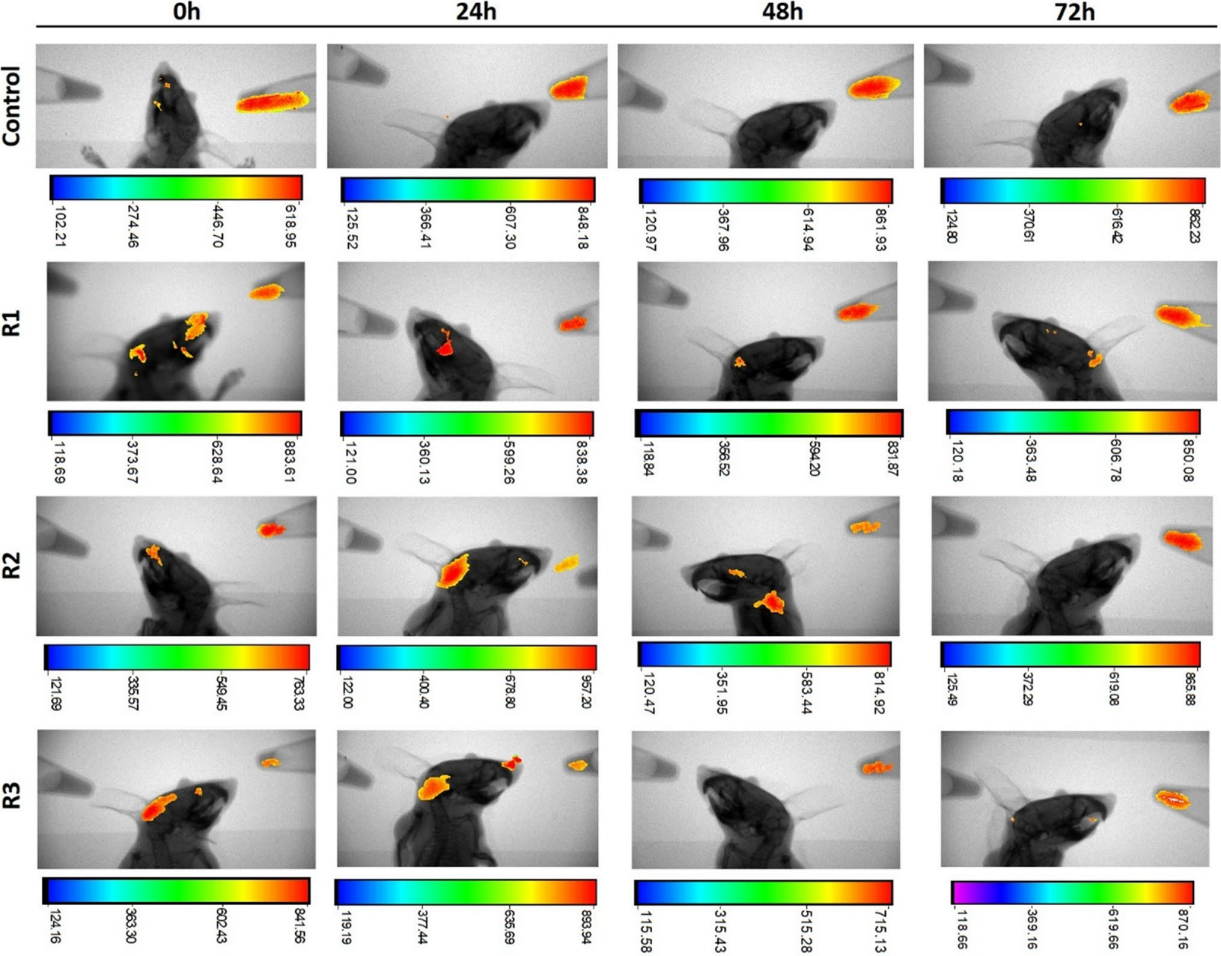
Time course in vivo imaging after intranasal inoculation of IRFP713-expressing *L. plantarum*. BALB/c mice (*n* = 3; R1-3) were inoculated intranasally with 4 × 10^9^ CFU of IRFP713-expressing *L. plantarum* 3.12.1. Control mice (*n* = 2) were inoculated with wild-type *L. plantarum* 3.12.1. Images are X-ray captions with IRFP713-derived signal fluorescence overlay obtained at emission/excitation wavelengths 650/700 nm. In vitro samples containing wild-type *L. plantarum* 3.12.1 and IRFP713-expressing *L. plantarum* 3.12.1 are shown on the left and on the right side of each caption, respectively. Color bars indicate fluorescence intensity. Images were obtained using the Bruker In Vivo (MS) FX PRO

Three mice were intranasally-administered 4 × 10^9^ CFU IRFP713-expressing *L. plantarum* 3.12.1 cells (1,268.57 mean fluorescence intensity, *n* = 3) to determine the time profile of the IRFP713 signal following inoculation of the bacteria. Two control mice were administered 4 × 10^9^ CFU of wild-type *L. plantarum* 3.12.1. (346.64 mean fluorescence intensity) similarly through the intranasal route. Fluorescence and X-ray images were captured immediately after bacterial inoculation (0 h) and on 24, 48, and 72 h, in both of mice and of the tubes containing the bacterial suspensions used for inoculation. Figure 5 shows the infrared fluorescence produced by the wild-type and IRFP713-expressing *L. plantarum* 3.12.1 at different time points, captured before the mice inoculation. After subtracting the non-IRFP713^+^-derived fluorescence (given by the wild-type fluorescence signal), stable expression of IRFP713 was observed in IRFP713^+^-*L. plantarum* 3.12.1 contained in the tube, during the time course of the experiment. Image captions of mice inoculated with IRFP713^+^-*L. plantarum* 3.12.1. (Fig. 6) show different bacterial localizations on the nasal cavity immediately after inoculation: the fluorescence signal was found on the nasal passages, the nasoturbinates and the nasopharyngeal duct. Despite some signal remaining on the nasal cavity, high fluorescence signal was observed in the pharynx of two mice at 24 h. After 48 h, fluorescence was observed in two of the three inoculated mice, suggesting bacterial localization on the nasopharyngeal duct and the pharynx. After 72 h, no fluorescence was observed in two out of the three mice, indicating elimination of the bacteria from the respiratory tract, possibly by secretion and dilution through the digestive tube. No significant fluorescence was detected in the control mice.

**Fig. 7:**
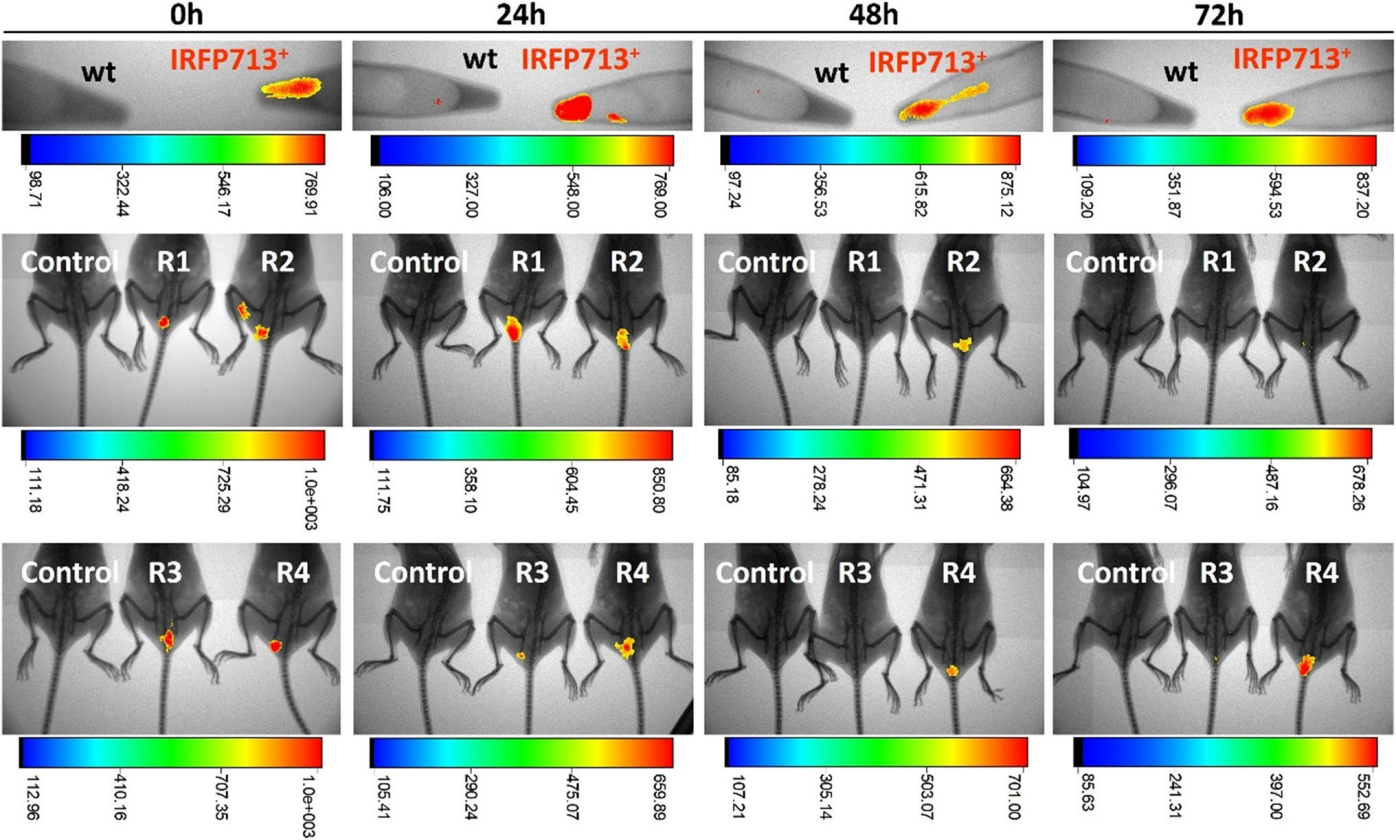
Time course in vivo imaging after intravaginal inoculation of IRFP713-expressing *L. plantarum*. BALB/c mice (*n* = 4; R1-4) were inoculated intravaginally with 5 × 10^9^ CFU of IRFP713-expressing *L. plantarum* 3.12.1. Control mice (*n* = 2) were inoculated with wild-type *L. plantarum* 3.12.1. Images are X-ray captions with IRFP713-derived signal fluorescence overlay obtained at emission/excitation wavelengths 650/700 nm. In vitro samples containing wild-type *L. plantarum* 3.12.1 and IRFP713-expressing *L. plantarum* 3.12.1 are shown on the left and on the right side of each caption, respectively, on the top panels. Color bars indicate fluorescence intensity. Images were obtained using the Bruker In Vivo (MS) FX PRO

Four mice were intravaginally inoculated with 5 × 10^9^ CFU of IRFP713-expressing *L. plantarum* 3.12.1 cells (947.61 mean fluorescence intensity, *n* = 3) to determine the time profile of the IRFP713 signal on the vaginal mucosa. In parallel, two control mice were administered 5 × 10^9^ CFU of wild-type *L. plantarum* 3.12.1 (514.91 mean fluorescence intensity). As before, fluorescence and X-ray images were captured immediately after bacterial inoculation (0 h) and at 24, 48, and 72 h, both in mice and in the tubes containing the bacterial suspensions used for inoculation. After subtracting the non-IRFP713^+^-derived fluorescence (given by the wild-type fluorescence signal), image captions of the four mice inoculated the IRFP713^+^-*L. plantarum* 3.12.1 showed bacterial location in the vaginal cavity at 0 and 24 h (Fig. 7). After 48 h, two out of the four mice showed fluorescence signal, indicating that bacteria remained in the vaginal cavity in these individuals. After 72 h, only one mouse appeared to remained colonized, suggesting bacterial secretion. No fluorescence was detected in the control mice.

## Discussion

In this work, stable and constitutive expression of IRFP713 was observed in three *L. plantarum* strains—*L. plantarum* NCDO 1193, *L. plantarum* ATCC 8014 and *L. plantarum* 3.12.1—through in vitro fluorescence microscopy, suggesting its potential as a reporter protein in these bacteria. IRFP713-expressing *L. plantarum* 3.12.1 was further imaged in live mice after intranasal and intravaginal inoculation using near-infrared fluorescence, allowing evaluation of its potential to colonize or reside in these mucosal sites using this non-invasive approach. Although other fluorescent *L. plantarum* strains have been built previously (Berlec et al. 2015; Salomé-Desnoulez et al. 2021), these have been used only for tracking these bacteria in the gastro-intestinal track of mice. This is the case of IRFP713, which open reading frame was cloned into different expression plasmids by Berlec et al. (2015) and used for the spatial location of *Lactococcus lactis*, *L. plantarum*, and *E. coli* in the gut of mice. Thus, to our knowledge, this is the first description of non-invasive in vivo imaging of lactiplantibacilli in the airways and vaginal mucosa of mice and the first description of using fluorescent lactiplantibacilli at these locations.

Albeit *L. plantarum* is commonly used as a vaginal probiotic (De Seta et al. 2014), the recent finding that this bacterium can be enriched in the healthy human nose and nasopharynx (de Boeck et al. 2020) suggests its usefulness as a vector for the delivery of prophylactic molecules also through the intranasal mucosa. In line with this, it is noteworthy to mention the recent work from Li et al. (2021) describing mucosal IgA responses against SARS-CoV-2 after intranasal administration of LP18:RBD in mice, a recombinant strain of *L. plantarum* expressing the receptor-binding domain (RBD) of the SARS-CoV-2 spike protein on its surface. The strain used for the expression of recombinant RBD—*L. plantarum* CGMCC 1.557 (also named LP18)—was originally obtained from the human gut, exhibiting probiotic properties with the potential for use in the production of probiotic fermented foods (Ren et al. 2014). Following inoculation, LP18:RBD elicited RBD-specific mucosal IgA antibodies in the respiratory and intestinal tract and a significant increase in CD^3+^ CD^4+^ T cells in the spleens of mice, suggesting triggering of humoral immune responses. However, in contrast with other recombinant *L. plantarum* mucosal vaccines (Kuczkowska et al. 2017; Oliveira et al. 2006), LP18:RBD was not able to elicit specific IgG antibodies, indicating poor immunogenicity beyond the mucosal (local) level. In this sense, the generation of effective IgG-based immunity could eventually benefit from formulating a vaccine with one or several *L. plantarum* strains obtained from the human respiratory mucosa or able to colonize this surface. After nasal delivery, these strains would possibly facilitate the heterologous proteins to be displayed for longer time to antigen presenting cells, allowing more efficient antigen uptake and stimulation of systemic immunity. For instance, assessing the colonization times of different *L. plantarum* in the mucosae using non-invasive in vivo imaging could be of special value ahead of testing the immunogenicity of aleatory strains at these sites.

The assessment of *L. plantarum* distribution in the vaginal and nasal mucosae of mice was intended for studying their relevancy against genitourinary infections in men and women and their potential as antigen delivery vectors at these locations, in the future. The selection of *L. plantarum* 3.12.1 for the in vivo imaging was based on our previous observations suggesting that this strain displays antimicrobial activity against *Neisseria gonorrhea* (data not published). As a result, residence times of *L. plantarum* 3.12.1 observed in live mice were found to be similar after intranasal and vaginal inoculation, varying from 24 to 72 h. The ability to temporarily colonize the vaginal mucosa of mice was somehow expected as the 3.12.1 strain was originally obtained from a vaginal swab of a healthy women. However, the capacity of this strain to withstand nasal clearance and at least temporarily colonize the nose of mice suggests that it may be useful as a mucosal bacterial vector both for vaginal and nasal use.

A linear relationship between fluorescence intensity and the lactiplantibacilli cell densities were observed for the three IRFP713^+^ bacterial strains, suggesting that fluorescence intensity could be used for bacterial quantification. However, the correlation between the fluorescence signal and the bacterial burden in vivo (CFU recovered from nasal and vaginal wash specimens) was not investigated, which should be addressed by using a larger number of animals for culturing inoculated bacteria. Opening of the inoculated cavities to confirm the bacterial localization by imaging should also be performed. Thus, the practical application of this technique needs further refinement.

Nisin is a bacteriocin synthetized and secreted by *Lactococcus lactis* subsp. *lactis*, which inhibits pathogenic food spoilage microorganisms such as *Listeria monocytogenes* and many other Gram-positive bacteria (Gharsallaoui et al. 2016). The products of the *nisRK* genes constitute a two-component regulatory system, which senses exogenous nisin and induces gene expression from the *nisA* promoter (P*nisA*) (Kuipers et al. 1995). However, due to the introduction of *nisRK* genes into the backbone of pNZ-IRFP713 (Berlec et al. 2015), constitutive expression of *irfp713* (loss of responsiveness to nisin) was observed in *L. plantarum* NCDO 1193 and ATCC 8014 strains. These results are in line with previous findings (Berlec et al. 2015) in which *L. plantarum* ATCC 8014 also lacked responsiveness to nisin. The exception was *L. plantarum* 3.12.1, in which slightly higher fluorescence intensity was observed in nisin-supplemented media when using fluorescence microscopy. On the other hand, nisin was found to have some inhibitory effect on the growth of *L. plantarum* ATCC 8014, causing a longer latency phase in the growth curve of this strain. This might have resulted in the lower fluorescence intensities observed for the first 20 h of growth in the presence of this compound, when compared to the cells grown in biliverdin-supplemented media. To circumvent this, lower nisin concentrations could be tested. According with these results, the addition of nisin (and its concentration range) should be individually considered for each *L. plantarum* strain in order to avoid any detrimental effect in the resulting fluorescence level. Due to higher fluorescence emission (compared to that of the background fluorescence in MRS), the chromophore biliverdin was shown to be necessary for fluorescence emission in the three bacterial strains tested, as expected.

As the fluorescence intensity and viability of the IRFP713-expressing *L. plantarum* strains were shown to be very high until days 28 and 60, respectively, it can be assumed that any gradual decrease in fluorescence during the time course of the animal experiments (up to 72 h) was likely due to bacterial secretion and not due to IRFP713 degradation or bacterial cell lysis. Moreover, the simultaneous imaging of the IRFP713^+^ cell suspensions contained within the microcentrifuge tubes showed no loss of fluorescence of the inoculated bacteria during the time course of the experiments. These observations suggest stable and constitutive expression of IRFP713 during this period, even in the absence of selective pressure. A possible limitation of using this technique would be the progressive loss of fluorescence if longer colonization times were observed (> 28 days). However, long-term lactiplantibacilli colonization may be hypothetically studied in the vaginal and nasal mucosa using a lactiplantibacilli strain expressing a chromosomally inserted *irfp713* gene.

Despite the need for further exploration, near-infrared fluorescence was shown to be a useful tool for the in vivo monitoring of *L. plantarum* distribution and persistence at the intranasal and intravaginal mucosal sites, through the stable and constitutive expression of IRFP713. In addition, the residence times observed for *L. plantarum* 3.12.1 in these locations suggests its potential for further applications as a mucosal vaccine vector.

## Supplementary Information

Below is the link to the electronic supplementary material.

Supplementary file1 (PDF 476 KB)

## Data Availability

The data that support the findings of this study are available from the corresponding author, upon reasonable request.
