# Supplementary material for: In vivo monitoring of Lactiplantibacillus plantarum in the nasal and vaginal mucosa using infrared fluorescence
Source: Appl Microbiol Biotechnol. 2022 Aug 24;106(18):6239–51. doi: 10.1007/s00253-022-12121-8 (PMC9398905; doi:10.1007/s00253-022-12121-8)
Supplement: Supplementary file 1 — Supplementary file1 (PDF 476 KB) [file 253_2022_12121_MOESM1_ESM.pdf]

Supplementary Information

Applied Microbiology and Biotechnology

In vivo monitoring of *Lactiplantibacillus plantarum* in the nasal and vaginal mucosa using infrared fluorescence

Sergio Silva-Bea[1], Mónica Francisco-Tomé[1], Jorge Cabrera[1,2], Carmen Potel[1,2], Maximiliano Álvarez[1,2], Sonia Pérez[1,2], Benito Regueiro[1,2] and Maria P. Cabral[1*]

[1]Group of Microbiology and Infectious Diseases, Galicia Sur Health Research Institute (IISGS), Vigo, Spain

[2]Microbiology Service, University Hospital of Vigo, Spain

***Correspondence:** clara.povoa@iisgaliciasur.es

Lactobacillus plantarum strain NBRC 15891 16S ribosomal RNA, partial sequence
Sequence ID: NR_113338.1Length: 1492Number of Matches: 1
Range 1: 4 to 348GenBankGraphics Next Match Previous Match

Alignment statistics for match #1

Score	Expect	Identities	Gaps	Strand
630 bits(341)	1e-180	343/345(99%)	0/345(0%)	Plus/Plus

```
Query  1    GAACGCTGGCGGCGTGCCTAATACATGCAAGTCGAACGAACTCTGGTATTGATTGGTGCT  60
            ||||||||||||||||||||||||||||||||||||||||||||||||||||||||||||
Sbjct  4    GAACGCTGGCGGCGTGCCTAATACATGCAAGTCGAACGAACTCTGGTATTGATTGGTGCT  63

Query  61   TGCATCATGATTTACATTTGAGTGAGTGGCGAACTGGTGAGTAACACGTGGGAAACCTGC  120
            ||||||||||||||||||||||||||||||||||||||||||||||||||||||||||||
Sbjct  64   TGCATCATGATTTACATTTGAGTGAGTGGCGAACTGGTGAGTAACACGTGGGAAACCTGC  123

Query  121  CCAGAAGCGGGGGATAACACCTGGAAACAGATGCTAATACCGCATAACAACTTGGACCGC  180
            ||||||||||||||||||||||||||||||||||||||||||||||||||||||||||||
Sbjct  124  CCAGAAGCGGGGGATAACACCTGGAAACAGATGCTAATACCGCATAACAACTTGGACCGC  183

Query  181  ATGGTCCGAGYTTGAAAGATGGCTTCGGCTATCACTTTTGGATGGTCCCGCGGCGTATTA  240
            |||||||||| |||||||||||||||||||||||||||||||||||||||||||||||||
Sbjct  184  ATGGTCCGAGNTTGAAAGATGGCTTCGGCTATCACTTTTGGATGGTCCCGCGGCGTATTA  243

Query  241  GCTAGATGGTGRGGTAACGGCTCACCATGGCAATGATACGTAGCCGACCTGAGAGGGTAA  300
            ||||||||||| ||||||||||||||||||||||||||||||||||||||||||||||||
Sbjct  244  GCTAGATGGTGGGGTAACGGCTCACCATGGCAATGATACGTAGCCGACCTGAGAGGGTAA  303

Query  301  TCGGCCACATTGGGACTGAGACACGGCCCAAACTCCTACGGGAGG  345
            |||||||||||||||||||||||||||||||||||||||||||||
Sbjct  304  TCGGCCACATTGGGACTGAGACACGGCCCAAACTCCTACGGGAGG  348
```

Figure S1. 16S rRNA sequencing results of *L. plantarum* 3.12.1.

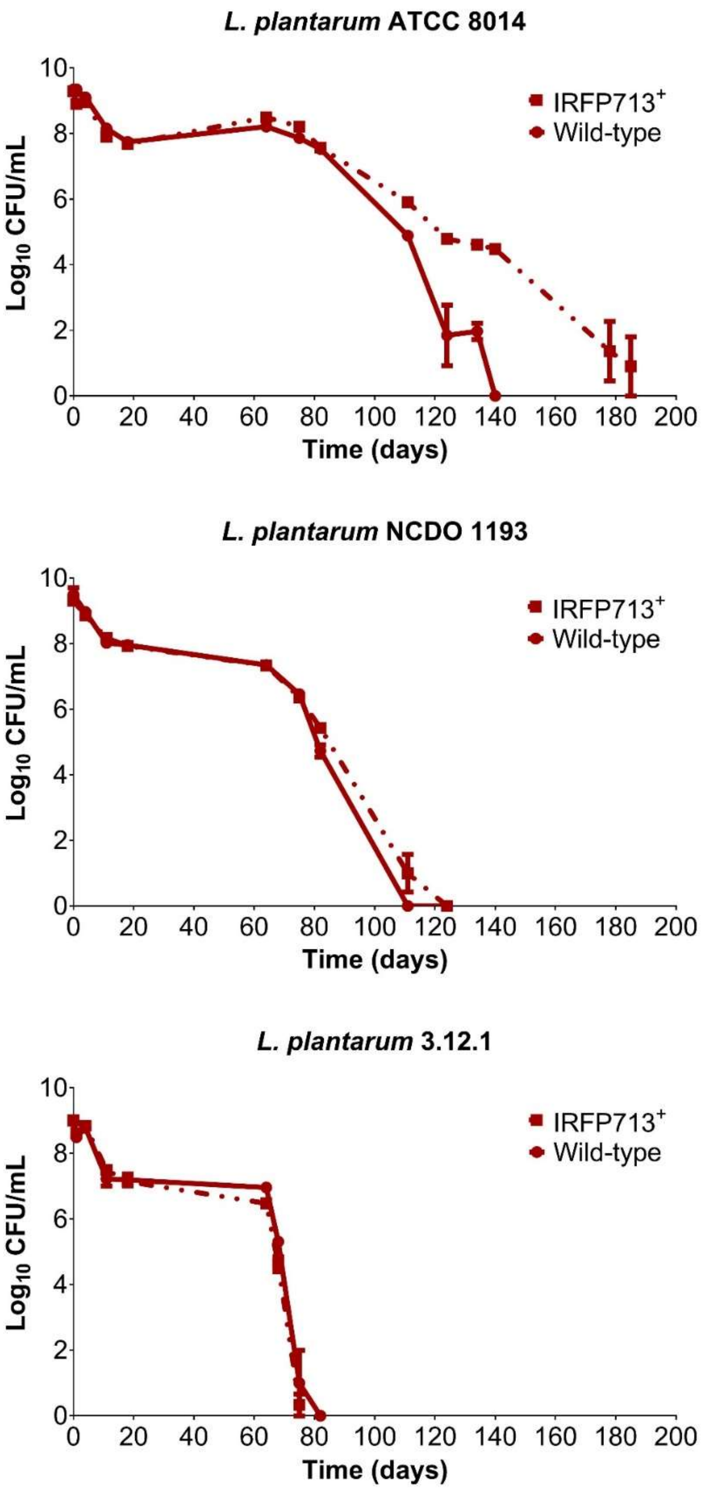

Figure S2. Viability of wild-type and IRFP-expressing *L. plantarum* ATCC 8014, NCDO 1193 and 3.12.1 strains during storage at 4 ºC. Overnight cultures of wild-type and pNZRK-IRFP713-harboring *L. plantarum* were used to inoculate fresh MRS (and MRS containing Cm) medium supplemented with biliverdin and nisin, incubated overnight, resuspended in sucrose and stored at 4 ºC until used for CFU determination. The data shown are mean ± SEM of 3 biological replicates.
